# Comparison of two techniques (in vivo and ex-vivo) for evaluating the elastic properties of the ascending aorta: Prospective cohort study

**DOI:** 10.1371/journal.pone.0256278

**Published:** 2021-09-13

**Authors:** Marie-Catherine Morgant, Siyu Lin, Diana Marin-Castrillon, Chloé Bernard, Aline Laubriet, Alexandre Cochet, Alain Lalande, Olivier Bouchot

**Affiliations:** 1 Department of Cardio-vascular and Thoracic Surgery, Dijon University Hospital, Dijon, France; 2 ImVia Laboratory, University of Burgundy, Dijon, France; 3 Department of Magnetic Resonance Imagery, Dijon University Hospital, Dijon, France; Stellenbosch University Faculty of Medicine and Health Sciences, SOUTH AFRICA

## Abstract

**Introduction:**

Aneurysms of the ascending aorta (AA) correspond to a dilatation of the ascending aorta that progressively evolves over several years. The main complication of aneurysms of the ascending aorta is type A aortic dissection, which is associated with very high rates of morbidity and mortality. Prophylactic ascending aorta replacement guidelines are currently based on maximal AA diameter. However, this criterion is imperfect. Stretching tests on the aorta carried out ex-vivo make it possible to determine the elastic properties of healthy and aneurysmal aortic fragments (tension test, resistance before rupture). For several years now, cardiac magnetic resonance imaging (MRI) has provided another means of evaluating the elastic properties of the aorta. This imaging technique has the advantage of being non-invasive and of establishing aortic compliance (local measurement of stiffness) without using contrast material by measuring the variation of the aortic surface area during the cardiac cycle, and pulse wave velocity (overall stiffness of the aorta).

**Materials and methods:**

Prospective single-center study including 100 patients with ascending aortic aneurysm requiring surgery. We will perform preoperative cine-MRI and biomechanical laboratory stretching tests on aortic samples collected during the cardiac procedure. Images will be acquired with a 3T MRI with only four other acquisitions in addition to the conventional protocol. These additional sequences are a Fast Low Angle Shot (FLASH)-type sequence performed during a short breath-hold in the transverse plane at the level of the bifurcation of the pulmonary artery, and phase-contrast sequences that encodes velocity at the same localization, and also in planes perpendicular to the aorta at the levels of the sino-tubular junction and the diaphragm for the descending aorta. For ex-vivo tests, the experiments will be carried out by a biaxial tensile test machine (ElectroForce®). Each specimen will be stretched with 10 times of 10% preconditioning and at a rate of 10 mm/min until rupture. During the experiment, the tissue is treated under a 37°C saline bath. The maximum elastic modulus from each sample will be calculated.

**Results:**

The aim of this study is to obtain local patient-specific elastic modulus distribution of the ascending aorta from biaxial tensile tests and to assess elastic properties of the aorta using MRI, then to evaluate the correlation between biaxial tests and MRI measurements.

**Discussion:**

Our research hypothesis is that there is a correlation between the evaluation of the elastic properties of the aorta from cardiac MRI and from stretching tests performed ex-vivo on aorta samples collected during ascending aorta replacement.

## Introduction

The thoracic aorta has a unique structural composition that enables it to withstand significant tissue stress mainly caused by blood pressure [1]. Despite their essential role in the cardiovascular system, the elastic properties of the aorta are still poorly known [2]. The artery wall is made up of three layers: the intima, the media and the adventitia. The intima (innermost layer) consists of a layer of endothelial cells, whose main role is to provide a thromboresistant surface. The media is a thick layer made up of a variable proportion of smooth muscle cells which play a role in arterial vasomotor response, elastic fibers (made up of an elastin core surrounded by microfibrils) which allow the distensibility of the arteries, and rigid collagen fibers (mostly type I and III). This layer plays an essential role in the resistance of tissues to tension [3]. The adventitia (outer layer) consists mainly of thick bundles of wavy collagen fibers (mostly of type I) [4]. This layer has good compliance for small strains but becomes stiff when there is a high level of strain [5]. The proportion of elastic fibers and collagen fibers varies even along the aorta with maximum compliance at its proximal portion (ascending aorta and aortic arch). The elastic properties of the arteries can change if the constitution of the artery wall changes. Indeed, when the elastin/collagen ratio decreases, the distensibility of the artery does too. Aneurysms of the ascending aorta are the result of a dilatation of the aorta that progressively increases over several years. The main complication of aneurysms of the ascending aorta is a type A aortic dissection (dissection hitting the ascending aorta). The course of acute type A aortic dissection is catastrophic—more than 50% of patients die during the acute phase if they are not treated surgically, and mortality ranges from 5 and 20% even with adequate surgical treatment [6–8]. The risk of aortic dissection is known to correlate with increased aorta diameter. In order to prevent this risk, the current guidelines recommend prophylactic replacement of the ascending aorta at 55 mm using a Dacron® graft (polyethylene terephthalate) [9], except for patients with connective tissue disorder (Marfan, Loeys-Dietz or Ehler-Danlos syndrome) or with bicuspid aortic valve. In these specific patients, the threshold is lower than that in other patients.

Yet some studies highlight that the aortic diameter of many patients with acute type A aortic dissection is smaller than 55 mm [10, 11]. The mechanisms and exact causes of dissection are still unclear. Though imperfect, the diameter of the aorta remains the only criterion to guide indications for prophylactic replacement of the ascending aorta. A better understanding of the biomechanical properties of the aorta could lead to the development of new, more effective criteria for preventing aneurysm complications.

Stretching tests performed on the aorta in a laboratory setting can be used to determine the elastic properties of healthy and aneurysmal aortic fragments (tension test, resistance before rupture) [12–14].

On the other hand, recent developments in cardiac magnetic resonance imaging (MRI) have made it possible to evaluate the elastic properties of the aorta as well, and MRI has demonstrated its robustness in assessing aortic stiffness. This imaging technique has the advantage of being non-invasive and of establishing without contrast material aortic compliance (local measurement), pulse wave velocity (global measurement) and also the overall stiffness of the aorta through the measurement of pulse wave velocity (PWV) [15–18]. The development of software for automatic detection of aortic contours also increases the reliability and reproducibility of the results [19]. However, studies have rarely attempted to establish a correlation between biomechanical data and magnetic resonance imaging data.

A better understanding the biomechanical properties of the aorta could lead to the development of biomechanical criteria and then to more accuracy for predicting the development of aortic aneurysm or aneurysm complications. The aim of this study is to obtain local patient-specific elastic modulus distribution of the ascending aorta from a biaxial tensile test and to assess elastic properties of the aorta using MRI. Then, we will evaluate whether there is a correlation between biaxial tests and MRI measurements.

## Materials and methods

### Ethics

The study has been approved by the national ethic committee *(Comité de Protection des Personnes)* (2018-A02010-55) and recorded on ClinicalTrials.gov (clinical registration number: NCT03817008). The French law does not require written consent for this study. According to ethics committee, all patients will receive written information note on the study. Oral consent will be obtained from the patient, with a written information note (explaining the principle and the course of the study) provided to the patient at the time of consent. An attestation of the patient’s oral consent will be signed by the investigator physician and countersigned by the patient.

**Fig 1:**
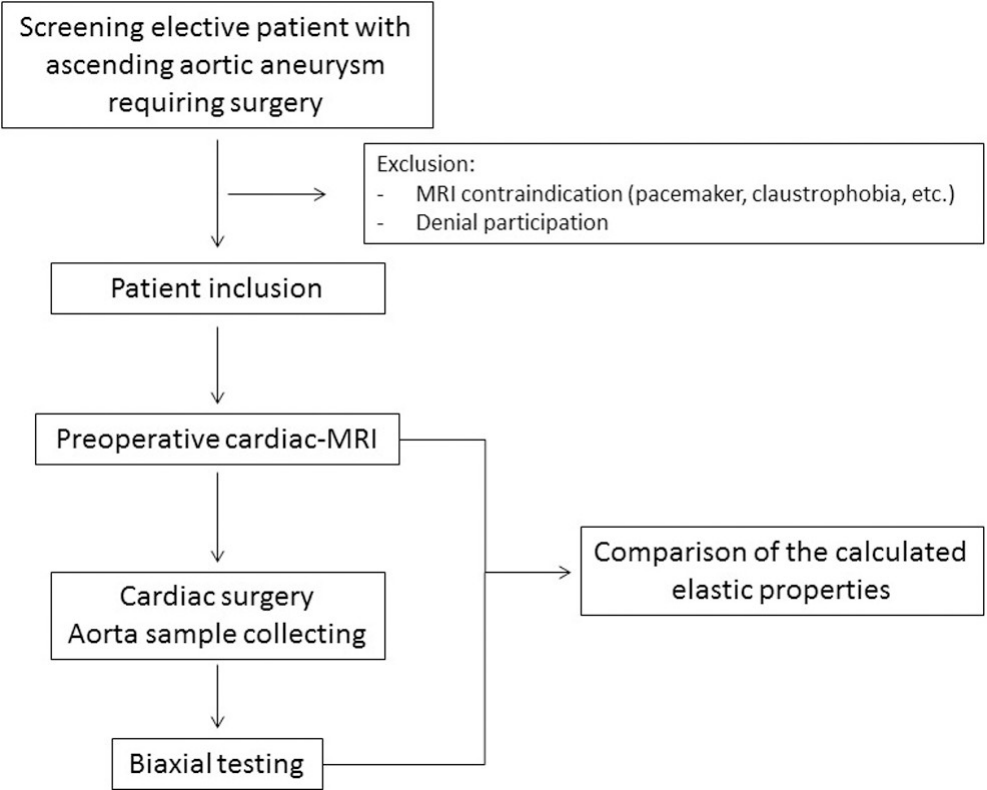
Flow chart of the study design.

The project is based on a prospective single-center study including 100 patients with ascending aortic aneurysm requiring surgery. We will perform preoperative cine-MRI and biomechanical laboratory stretching tests on aortic samples collected during the cardiac procedure (Fig 1). The aneurysmal portion of the ascending aorta will be resected and replaced by a Dacron® graft (polyethylene terephthalate). We will exclude elective patients who did not have preoperative MRI (potentially due to MRI contraindications such as cardiac pacemaker or claustrophobia).

Statistical analysis will be performed to establish whether there is a correlation between MRI compliance measures and laboratory aortic elasticity tests.

### Cardiac-MRI and image processing

**Fig 2:**
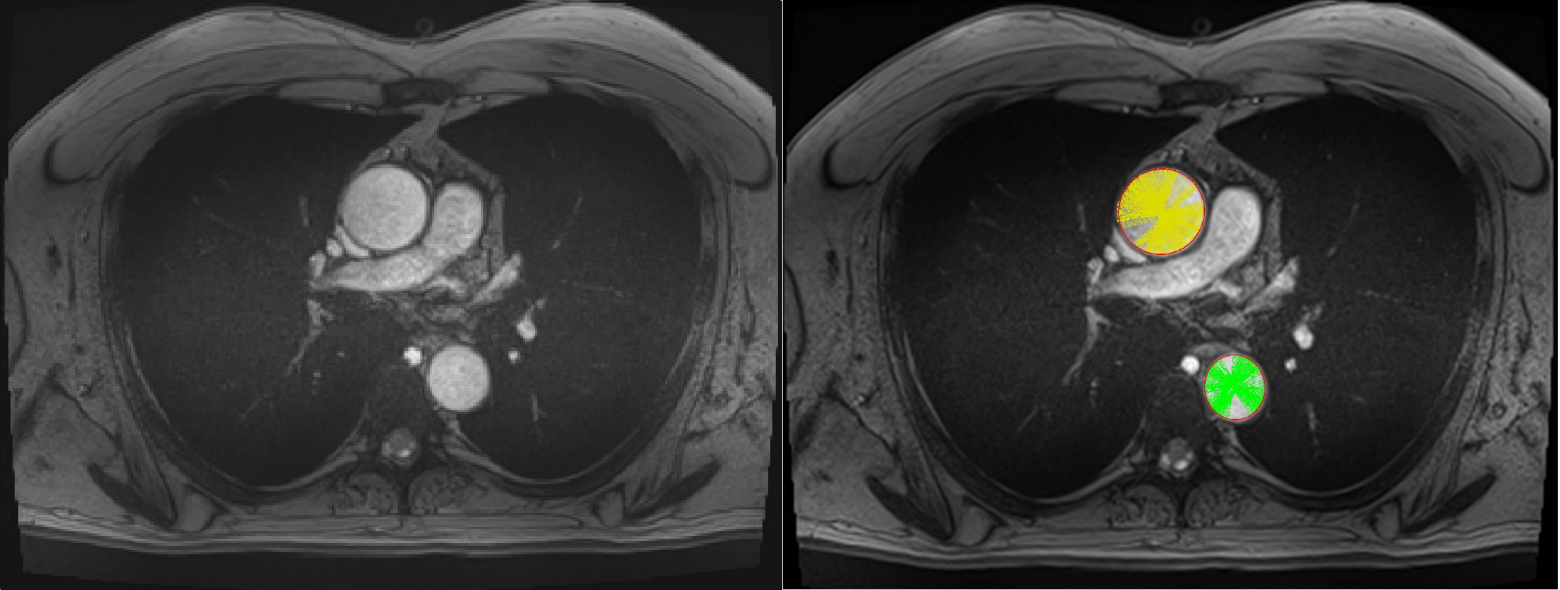
Automatic detection of the contours of the ascending (in green) and descending (in yellow) aortas before surgery.

**Fig 3:**
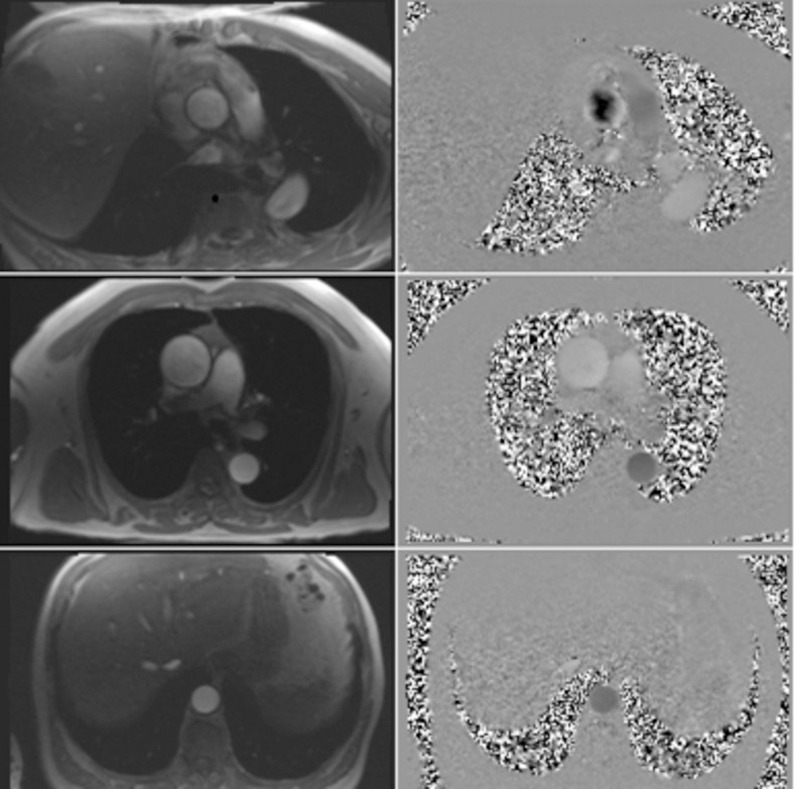
Phase-contrast sequences.

Cardiac MRI will be used to assess aortic compliance and pulse wave velocity (PWV) before the cardiac procedure. Images will be acquired with a 3T MRI (Skyra, Siemens Healthineers, Erlangen, Germany), with only four other acquisitions in addition to the conventional protocol (one cine-MRI (Fig 2) and three phase-contrast sequences that encodes velocity (Fig 3), that correspond to four additional breath-holds). The conventional protocol include a series of T1-weighted Turbo Spin Echo images in oblique-sagittal orientation that cover the aorta, cine-MRI in oblique sagittal and oblique coronal LVOT (Left ventricular outflow tract) planes, as in sinus plane, and an angio-MRI (for total duration of around 20 minutes). Firstly, a Fast Low Angle Shot (FLASH)-type sequence will be performed during a short breath-hold in the transverse plane at the level of the bifurcation of the pulmonary artery. This sequence is preferred to a Steady State Free Precession (SSFP)–type sequence because it is less sensitive to noise due to rapid or turbulent flow at 3T. This plane will allow operators to analyse the ascending and descending aorta simultaneously. Acquisition with retrospective electrocardiogram-gating will provide images at all the different phases of the cardiac cycle with a temporal resolution between 20 msec and 34 msec, echo time of 3.42 msec, repetition time of 7.21 msec, flip angle of 12°, spatial resolution between 1.09 × 1.09 mm²/pixel and 1.25 × 1.25 mm²/pixel (corresponding to a field of view varying from 350 mm to 400 mm) and slice thickness of 5 mm. About 35 images cover the cardiac cycle. Generalized autocalibrating partially parallel acquisitions (GRAPPA) with an acceleration factor of 2 will be performed. Distortion correction and a pre-scan normalized filter were also used.

For each cardiac phase, the contours of the ascending and descending aorta will be automatically detected with a home-made software based on the method described by Mitéran et al [19]. This automatic method is based on an adaptation of a curvilinear region detector. Near circular regions were detected (ascending and descending aorta cross-sections) in each image of the sequence using robust scale-space based method with removing of false positives using probabilistic approach. On each image, the cross-sectional area will be calculated from the detected contour (Fig 2). The minimal and maximal areas of the ascending and descending aorta will be then recorded. The method is completely automatic and no external intervention will be required by the operator. If the contours are not detected by the software because of artifacts, the patient will be excluded to the study in order to avoid biases due to operator interference. Brachial blood pressure will be measured with an MRI-compatible arm blood pressure cuff and automated blood pressure monitor (non-invasive technique).

Secondly, for the PVW study, phase-contrast sequence that encodes velocity will be acquired at the same plane as the cine images, and also in planes perpendicular to the aorta at the levels of the sino-tubular junction and the diaphragm for the descending aorta. The velocity encoding gradient will be set in through plane direction with a variable VENC (maximum encoding velocity) according to the patient. The following parameters will be used (GRE-based sequence): echo time [TE] of 2.47 ms, repetition time of 9.28 msec, flip angle of 20, slice thickness of 6 mm, spatial resolution between 1.78 mm²/pixel and 2.08 mm²/pixel (corresponding to a field of view varying from 350 mm to 410 mm). Distortion correction was used on these images. Thirty to 50 images cover the cardiac cycle (according to the patient) with retrospective electrocardiogram-gating. The slope at the beginning of the systolic phase will be used for the estimation of the time difference between the arrival of pulse waves at two different levels of the aorta. The length of the aortic segments will be calculated from the anatomic SSFP slices. The centre of the aorta will be indicated on each slice between two levels of interest.

Aortic compliance and pulse wave velocity will be calculated from Eqs (1) and (2):

$$Compliance\;(mm^{2}/mmHg)=\frac{Maximal\;area-Minimal\;area}{Systolic\;pressure-Diastolic\;pressure}$$ (1)

$$Pulse\;Wave\;Velocity\;(mm/s)=\frac{Arterial\;path\;length}{Arterial\;pulse\;transit\;time}$$ (2)

### Bi-axial testing

**Fig 4:**
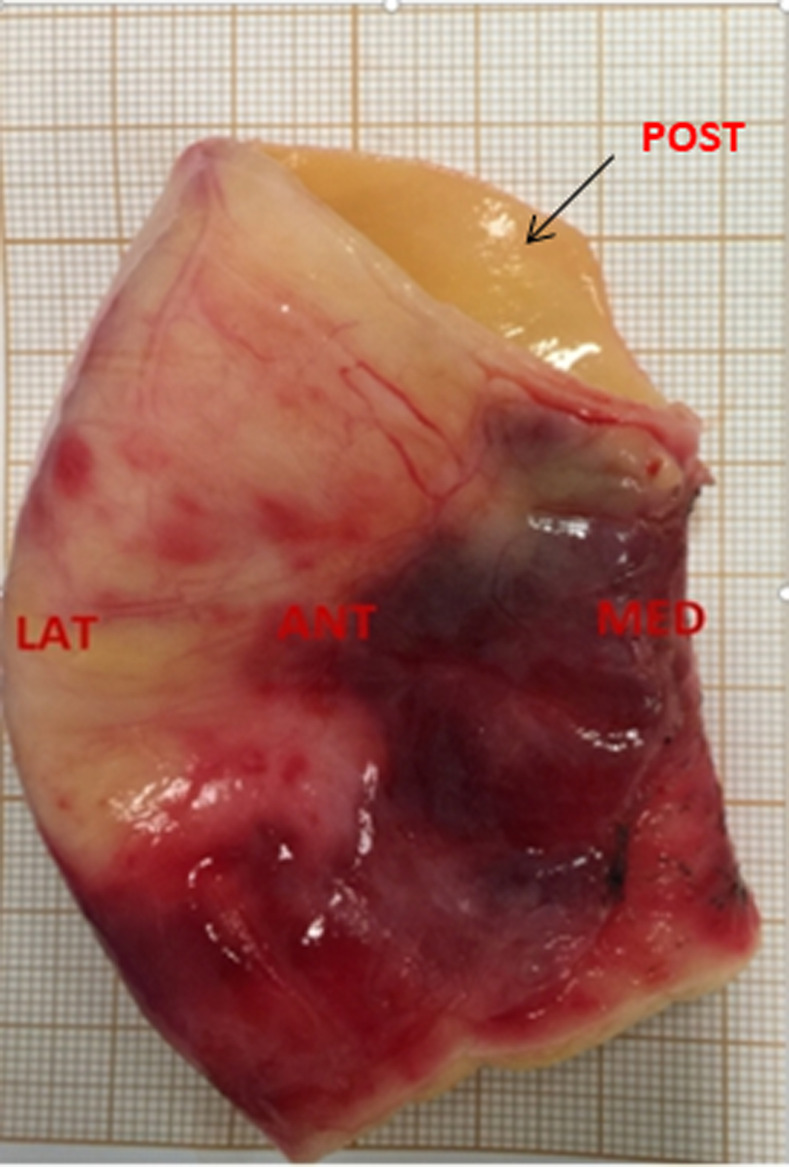
Ascending aorta sample collected during cardiac surgery.

Bi-axial tests will be performed on the surgical sample collected during the ascending aorta replacement procedure. The surgical specimen collected during the procedure will be placed in a preservative liquid (phosphate buffered saline: PBS), then immediately transferred to the laboratory (Fig 4). Testing will be done within 6 hours of tissue collection. The timeline of the tissue collection will be as follows: T0: tissue is replaced in from the patient; within T0+40 min: tissue is transferred to the laboratory where biaxial tensile test will be perform in PBS (phosphate buffered saline) in a cooler bag to avoid temperature variations; within T0+1h: tissue is cut based on the quadrants with the measurement of thickness; within T0+3h-6h (depend on the sample size): the biaxial tensile test is completed. During the time, the under-tested specimens are kept in the 4 C fridge under PBS.

All samples will be collected as intact rings, with orientation marked by a single clip.

**Fig 5:**
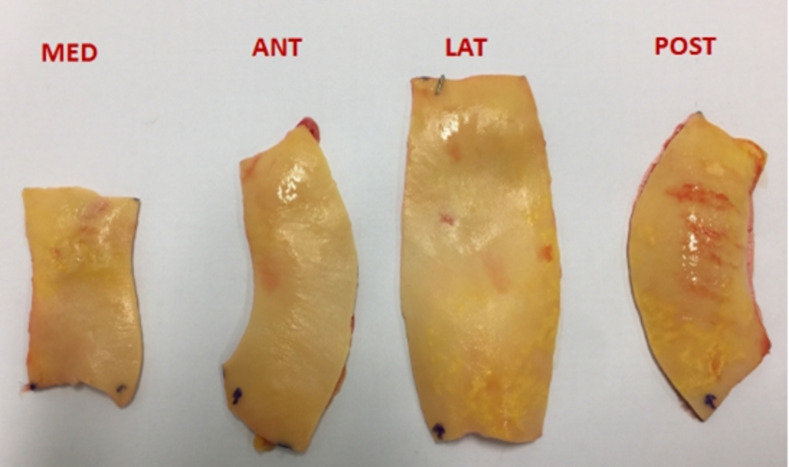
Partition of aorta sample to four quadrants.

**Fig 6:**
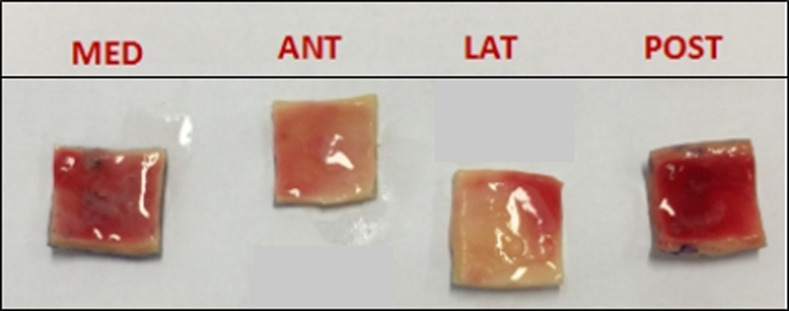
Section of aorta sample with square shape (size: 15 mm * 15 mm). One specimen by quadrant.

Pathologic ascending aortic tissue samples will be obtained from patients undergoing elective surgical replacement of the ascending aorta. All the aortic wall samples will be partitioned relative to medial (MED), posterior (POST), lateral (LAT), and anterior (ANT) quadrants (Fig 5). Each ascending aorta sample will be cut into a square shape (15 mm x 15 mm) with marking the circumferential and longitudinal directions (Fig 6). For each specimen, average thickness will be measured with an electronic micrometer (Litematic VL-50, Mitutoyo®). The experiments will be carried out by a biaxial tensile test machine (ElectroForce®). Each specimen will be stretched with 10 times of 10% preconditioning and at a rate of 10 mm/min until rupture. During the experiment, the tissue is treated under a 37°C saline bath [20, 21].

Young’s modulus (E) is a measure of the ability of a material to withstand changes in length (strain) when under lengthwise tension or compression (tensile stress). Maximum elastic modulus calculation was previously described by Duprey et al [20].

Briefly, we assume that the aortic wall is an incompressible material. Stress *σ* is defined by Eq (3):

$$Tensile\;Stress\left(\sigma (\varepsilon )\right)(Pa)=\frac{F}{A}$$ (3)

F is the load and A the cross sectional area in mm². Strain is defined by Eq (4):

$$Strain(\varepsilon )=\frac{\Delta L}{L}$$ (4)

ΔL represents the instantaneous stretch and L represents the length of the specimen. Sometimes referred to as the modulus of elasticity, Young’s modulus is directly related to the longitudinal stress divided by the local strain (local relative displacement) and can be derived using Eq (5):

$$Young^{\prime }s\;modulus(E)(Pa)=\frac{Tensile\;Stress\;(\sigma (\varepsilon ))}{Strain\;(\varepsilon )}$$ (5)

**Fig 7:**
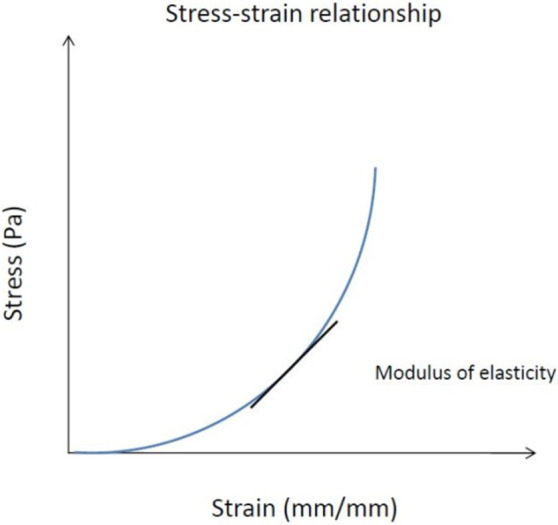
The stress-strain relationship.

The maximum elastic modulus from each sample will be taken from each curve as the slope of the tangent of the stress-strain relationship (Fig 7).

### Statistical analysis

The analyzed variables are continuous variables that will be expressed by their mean and standard deviation. We will test the normality of the variables with the Skewness and Kurtosis tests. STATA 14 statistical software (StataCorp, LP, College Station, Texas, and United States) will be used for the analyses. Continuous data will be analysed using a paired t-test or Wilcoxon test when variables will not follow a normal distribution. To study the correlation between MRI measurements and laboratory tests, we will use the parametric tests of correlation. In the absence of normality, we will use Spearman’s nonparametric test. Then we will use a univariate and multivariate linear regression model with the dependent variable being the MRI measurement. With a sample size of 100 patients, for an hypothesis H_0_: ρ = 0.4 and an hypothesis H_1_: ρ = 0.6, the statistical power is 0.84.

## Discussion

Preliminary study about the aortic compliance evaluation from MRI in pre- and post-operative ascending aortic replacement surgery shown that the aortic root replacement by graft was not associated with changes in elastic properties of the descending aorta at short term [22], However, for the ascending aorta, we found on a short sample that a local variability could happen in the aortic stiffness between different quadrants [23].

In case of positive correlation between compliance and/or PWV of the aorta measured by cardiac-MRI and elasticity testing, it would demonstrate the robustness of cardiac MRI for the assessment of the elastic properties of the aorta, even if the compliance is a local parameter that only accounts for in-plane area change at a specific localization. It will be then be necessary to determine the risk thresholds for aortic wall rupture, which is responsible for aortic dissections. This could be used to monitor aneurysms of the ascending aorta or patients without dilatation but with a family member with an aneurysm or aortic dissection. Although the experiments will be performed under different settings (parameters obtained in-vivo from MRI versus ex-vivo evaluation for elastic testing), the results could conclude that cardiac MRI could also guide surgical indications for replacement of the ascending aorta based on patient-specific biomechanical criteria rather than absolute diameter measurements that do not consider the specific characteristics of each patient’s artery walls. The potential heterogeneity of the cohort could be a limitation but the size of the considered cohort should reduce this bias.

## Supporting information

S1 File Consent English version. (PDF)

S2 File Consent French version. (PDF)

S3 File Note information English version. (PDF)

S4 File Note information French version. (PDF)
