# Supplementary material for: Comparison of two techniques (in vivo and ex-vivo) for evaluating the elastic properties of the ascending aorta: Prospective cohort study
Source: PLoS One. 2021 Sep 13;16(9):e0256278. doi: 10.1371/journal.pone.0256278 (PMC8437267; doi:10.1371/journal.pone.0256278)
Supplement: S1 File — (PDF) [file pone.0256278.s001.pdf]

Express Informed Consent

Prospective evaluation of the biomechanical properties of the thoracic aorta using magnetic resonance imaging and in-vitro elasticity tests in patients with ascending aortic aneurysms.

Study MECATHOR 2018-A02010-55

(Done in 2 copies: one copy is given to the participant, the other is kept by the investigator)

The Investigating Physician of the Cardiovascular and Thoracic Surgery Department proposed me to participate in the research involving the human person MECATHOR, of which CHU DIJON BOURGOGNE is promoter.

I was informed of the objective and the methods of carrying out this research involving the human person as well as my conditions of participation, my rights, the expected benefits, the constraints and the foreseeable risks, and I obtained the answers to the questions I asked.

I have read the information document that was explained to me and I will keep a copy of it.

I declare on my honour that I am affiliated to or a beneficiary of a social security scheme.

I accept for reasons related to my safety and for the proper conduct of research involving humans:
- To answer the questions I will be asked about my medical history and to follow all the instructions and directions that will be given to me by the medical investigator or his team, including those detailed in the information document.
- To contact the investigating physician or his or her team as soon as possible if I present an abnormal event.

I also agree :
- That my entire medical file be consulted by the persons authorized in the context of this research.
- The collection of the medical and personal data described in the information document as well as their computer processing by the promoter or by structures acting on his behalf.

I have noted that :
- The contact details of the investigating physician are noted on the information note that was given to me.
- My participation in this study is voluntary and I may at any time decide to interrupt my participation without justification and without affecting the quality of the care I will receive. I understand that in case of withdrawal of consent, my previously collected data may not be deleted and may continue to be processed under the conditions provided for by the research.
- All costs related to the research will be borne by the sponsor.
- My consent does not relieve the investigator and the sponsor of their responsibilities to me.

I freely and voluntarily agree to participate in the research proposed to me.

Attestation of oral consent of the patient

Prospective evaluation of the biomechanical properties of the thoracic aorta using magnetic resonance imaging and in-vitro elasticity tests in patients with ascending aortic aneurysms.

Study MECATHOR – 2018-A02010-55

(Done in 2 copies: one copy is given to the participant, the other is kept by the investigator)

I informed the patient of the objective and modalities of this research as well as the conditions of participation, his rights, expected benefits, constraints and foreseeable risks.

The patient read the information document that was explained to him and I gave him a copy.

The patient obtained the answers to the questions asked.

Oral consent :

Patient surname and first name : …………………………………………………………

Date of oral consent: ………/………/………

**To be completed by the investigating physician**<br><br>**I, the undersigned, (surname-first name)…………………………………………………………**<br><br>**declare that I have obtained the patient's oral consent to participate in this research.**	Signature<br><br><br><br>Date ……/………/…….
