# Supplementary material for: Comparison of two techniques (in vivo and ex-vivo) for evaluating the elastic properties of the ascending aorta: Prospective cohort study
Source: PLoS One. 2021 Sep 13;16(9):e0256278. doi: 10.1371/journal.pone.0256278 (PMC8437267; doi:10.1371/journal.pone.0256278)
Supplement: S2 File — (PDF) [file pone.0256278.s002.pdf]

Consentement Eclairé Exprès

Evaluation prospective des propriétés biomécaniques de l'aorte thoracique couplant imagerie par résonance magnétique et tests in-vitro d'élasticité chez des patients présentant un anévrisme de l'aorte ascendante

Etude MECATHOR

+ N° d'enregistrement

(Fait en 2 exemplaires : un exemplaire est remis au participant, l'autre est conservé par l'investigateur)

Le Médecin investigateur du service de chirurgie cardio-vasculaire et thoracique m'a proposé de participer à la recherche impliquant la personne humaine MECATHOR, dont le CHU DIJON BOURGOGNE est promoteur.

J'ai été informé(e) de l'objectif et des modalités de réalisation de cette recherche impliquant la personne humaine ainsi que de mes conditions de participation, de mes droits, des bénéfices attendus, des contraintes et des risques prévisibles, et **j'ai obtenu les réponses** aux questions que j'ai posées.

J'ai pris connaissance du document d'information qui m'a été expliqué et **j'en conserverai** un exemplaire.

Je déclare sur l'honneur être affilié(e) à un régime de sécurité sociale ou bénéficiaire d'un tel régime.

J'accepte pour des raisons liées à ma sécurité et pour le bon déroulement de la recherche impliquant la personne humaine :

- De répondre aux questions qui me seront posées à propos de mes antécédents médicaux et de suivre toutes les consignes et instructions qui me seront données par le médecin-investigateur ou son équipe, dont celles qui sont détaillées dans le document d'information.
- De contacter le médecin-investigateur ou son équipe dans les délais les plus brefs si je présente un événement anormal.

J'accepte également :

- Que l'ensemble de mon dossier médical soit consulté par les personnes habilitées dans le cadre de cette recherche.
- Le recueil des données médicales et personnelles décrites dans le document d'information ainsi que leur traitement informatique par le promoteur ou par des structures agissant pour son compte.

J'ai noté que :

- Les coordonnées du médecin-investigateur sont notées sur la note d'information qui m'a été remise.
- Ma participation à cette étude est volontaire et je peux à tout moment décider d'interrompre ma participation sans justification et sans que cela influence la qualité des soins que je recevrai. J'ai compris qu'en cas de retrait de consentement, mes données recueillies antérieurement pourront ne pas être effacées et pourront continuer à être traitées dans les conditions prévues par la recherche.
- L'intégralité des frais liés à la recherche seront pris en charge par le promoteur.
- Mon consentement ne décharge pas l'investigateur et le promoteur de leurs responsabilités à mon égard.

J'accepte librement et volontairement de participer à la recherche qui m'est proposée.

Attestation d'obtention du consentement oral du patient

Evaluation prospective des propriétés biomécaniques de l'aorte thoracique couplant imagerie par résonance magnétique et tests in-vitro d'élasticité chez des patients présentant un anévrisme de l'aorte ascendante

Etude MECATHOR

+ N° d'enregistrement

(Fait en 2 exemplaires : un exemplaire est remis au participant, l'autre est conservé par l'investigateur)

J'ai informé le patient de l'objectif et des modalités de réalisation de cette recherche ainsi que des conditions de participation, de ses droits, des bénéfices attendus, des contraintes et des risques prévisibles.

Le patient a pris connaissance du document d'information qui lui a été expliqué et **je lui en ai remis** un exemplaire.

Le patient a obtenu les réponses aux questions posées.

<table>
<tr><td colspan="2"><u>Consentement oral</u>

Nom-prénom du patient : ……………………………………………………….

Date de l'obtention du consentement oral : ………/………/………</td></tr>
<tr><td>A compléter par le médecin-investigateur

Je, soussigné(e), (nom-prénom) ……………………………………………………….

déclare avoir obtenu du patient le consentement oral à la participation à cette recherche</td><td>Signature

Le………/………/……….</td></tr>
</table>
