# Supplementary material for: Comparison of two techniques (in vivo and ex-vivo) for evaluating the elastic properties of the ascending aorta: Prospective cohort study
Source: PLoS One. 2021 Sep 13;16(9):e0256278. doi: 10.1371/journal.pone.0256278 (PMC8437267; doi:10.1371/journal.pone.0256278)
Supplement: S3 File — (PDF) [file pone.0256278.s003.pdf]

IM-2016-0369-002

Information note

Prospective evaluation of the biomechanical properties of the thoracic aorta using magnetic resonance imaging and in-vitro elasticity tests in patients with ascending aortic aneurysms.

Study MECATHOR

N° : 2018-A02010-55

(Done in 2 copies: one copy is given to the participant, the other is kept by the investigator)

Promoteur : CHU Dijon Bourgogne – Délégation à la Recherche Clinique et à l'Innovation
1, Bd Jeanne d'Arc
BP 77908 - 21079 Dijon Cedex
Tél : 03 80 29 50 15 / Fax : 03 80 29 36 90

Investigating Physician :

Degre, Name, First name:
Name of department:
Name of the institution:
Address:
Phone number:

and the CHU Dijon Bourgogne are currently conducting a study on ascending aortic aneurysms, coordinated by Dr. Marie-Catherine MORGANT of the CHU Dijon Bourgogne.

We would like to ask you to participate in an interventional research with minimal risks and constraints, carried out according to the law n° 2012-300 of March 5, 2012 relating to the public health policy, modified by the ordinance n°2016-800 of June 16, 2016 and its application decrees, and defined in the 2° of the article L1121-1 of the public health code.

We present here the information necessary to understand the interest and the course of the study, the expected benefits, the constraints and the foreseeable risks.

Your participation in this research is entirely voluntary and you have the right to refuse to participate. In this case, you will continue to benefit from the best possible medical care, according to current knowledge.

Read this leaflet carefully, it belongs to you.
You can pass it on and talk to your doctor or relatives for advice.
Ask any questions you think may be useful.
Once you have received satisfactory answers to your questions and have had a suitable period of time to think about it, you can then decide whether you want to take part in this study.

Why this research?

A thoracic aortic aneurysm is a localised dilation of the part of the aorta between its origin in the heart and the diaphragm, the horizontal muscle that separates the rib cage from the abdomen.

Although the mechanisms underlying the development of an aneurysm are fairly well known, we still do not know why some aneurysms rupture when they are still small (< 50 mm), while others exceed this diameter without rupturing.

The only curative treatment is surgical, with replacement of the dilated portion by prosthesis. Currently, surgical recommendations are based on the maximum diameter of the ascending aorta (between 45 and 55 mm depending on the existence of any associated risk factors for rupture (connective tissue disease, bicuspid aortic valve, rapid progression of the aneurysm)).

What is the aim of this research?

To better understand these phenomena, we want to study the elastic properties of the aortic wall from your cardiac MRI images (computer image processing). In parallel, we will directly measure the elasticity of the aorta on the operating room, i.e. on the portion of the diseased aorta that will be removed and replaced by a prosthesis during the surgery (ex-vivo test).

→ How you will participate in this study:

Pre-operative consultation or arrival in the cardiovascular and thoracic surgery department for ascending aorta surgery

Information on the principle and progress of the study - Delivery of the written information note

↓

Analysis of pre-operative cardiac MRI images

Retrieval of your pre-operative cardiac MRI images and image analysis

↓

Surgery of the ascending aorta

Collection of the surgical specimen (aneurysmal aorta wall)
Performing of ex-vivo elasticity tests on the surgical specimen

↓

End of your participation in the study

- Cardiac MRIs are tests that are routinely ordered before and after an operation on the ascending aorta. Therefore, you do not have to perform any additional imaging tests.
- The wall of the aorta studied is a surgical specimen, i.e. it is normally destined for disposal once your operation is complete. In no case will we take more aorta than your pathology requires. Elasticity measurements will be performed on 4 fragments taken from this surgical specimen.
- The imaging analyses will be performed on the preoperative MRI examinations that you will perform as part of your normal management.
- The elasticity analyses on the wall of the aorta will be carried out exclusively on the surgical specimen, i.e. on the part of your aorta destined for disposal. We will not remove more wall than your pathology requires.
- In this study, general epidemiological and medical data, preoperative and postoperative cardiac MRI imaging data with specific elasticity analysis and in-vitro data of elasticity measurements of your aortic wall will be collected.

What are the possible benefits and risks ?

No direct benefit to yourself is expected, but there is a potential collective benefit in the medium term depending on the results. If MRI measurements of aortic compliance prove to be correlated with biomechanical measurements, this would allow adaptation of operative indications according to the specific imaging data of aortic compliance for each patient. Moreover, this study does not present any additional risk since there is no additional procedure compared to usual practice.

What are the medical management methods ?

This research does not modify at any time the modalities of your care and does not impose any follow-up at the end of the research or in case of premature termination of the research, as your participation ends at the end of your first post-operative follow-up.

The regulatory framework

This study is carried out in accordance with Law n° 2012-300 of 5 March 2012, known as the "Jardé Law" relating to public health policy, amended by Ordinance n°2016-800 of 16 June 2016 and its implementing decrees, and defined in 2° of Article L1121-1 of the Public Health Code.

The promoter has taken out civil liability insurance to cover the possible harmful consequences of this study (company SHAM, contract number: 129.234, 18, rue Edouard Rochet, 69372 LYON Cedex 08).

This study has received a favourable opinion for its implementation from the Comité de Protection des Personnes (CPP) Sud-Est IV, dated 16/10/2018.

Protection of your data

This study will be carried out in accordance with the French law n°78-17 of 6 January 1978 on data processing, files and freedoms, as amended, and with the General Regulation on the Protection of Personal Data (RGPD), adopted at the European level, which came into force on 25 May 2018.
The medical and personal data concerning you and those associated with your samples will be processed electronically in order to establish the results of the study, in accordance with the exceptions provided for in Article 9 of the RGPD allowing the processing of health data.
This processing will be confidential as your data will only be identified by your initials associated with a code number. It will be transmitted to the Research Sponsor and kept for 15 years.
Furthermore, in the event of withdrawal of consent, your previously collected data will be used and may be processed in the conditions provided for in the research.

Nevertheless, you have several rights that you can exercise by writing to the Data Protection Officer - CHU Dijon Bourgogne - 1 Bd Jeanne d'Arc - 21079 DIJON Cedex: the right to access and rectify your data, the right to limit their computerised processing, the right to object to their transmission, the right to be forgotten (deletion of your data), and the right to lodge a complaint with the CNIL (Commission nationale de l'information et des libertés)

Your rights regarding your participation in this study:

- At any time during the study, you can contact the physician-investigator for any additional information about the study, your participation or your personal data related to your health.
- If you wish, you may be informed of the overall results of the research at the end of the study by the physician-investigator.
- The results of this study may be the subject of communications and/or publications in scientific journals in which your name will never be used.

Your participation is voluntary and free

Your possible refusal to participate will have no consequences on the type and quality of your treatment, or on your relationship with your doctor. If you agree to participate, you can leave this study at any time without justification or consequence on the quality of your care. You just need to inform your investigating doctor.

We thank you for your cooperation.
If you agree to participate in this study, we ask you to give your oral consent.

Express Informed Consent

Prospective evaluation of the biomechanical properties of the thoracic aorta using magnetic resonance imaging and in-vitro elasticity tests in patients with ascending aortic aneurysms.

Study MECATHOR 2018-A02010-55

(Done in 2 copies: one copy is given to the participant, the other is kept by the investigator)

The Investigating Physician of the Cardiovascular and Thoracic Surgery Department proposed me to participate in the research involving the human person MECATHOR, of which CHU DIJON BOURGOGNE is promoter.

I was informed of the objective and the methods of carrying out this research involving the human person as well as my conditions of participation, my rights, the expected benefits, the constraints and the foreseeable risks, and I obtained the answers to the questions I asked.

I have read the information document that was explained to me and I will keep a copy of it.

I declare on my honour that I am affiliated to or a beneficiary of a social security scheme.

I accept for reasons related to my safety and for the proper conduct of research involving humans:
- To answer the questions I will be asked about my medical history and to follow all the instructions and directions that will be given to me by the medical investigator or his team, including those detailed in the information document.
- To contact the investigating physician or his or her team as soon as possible if I present an abnormal event.

I also agree :
- That my entire medical file be consulted by the persons authorized in the context of this research.
- The collection of the medical and personal data described in the information document as well as their computer processing by the promoter or by structures acting on his behalf.

I have noted that :
- The contact details of the investigating physician are noted on the information note that was given to me.
- My participation in this study is voluntary and I may at any time decide to interrupt my participation without justification and without affecting the quality of the care I will receive. I understand that in case of withdrawal of consent, my previously collected data may not be deleted and may continue to be processed under the conditions provided for by the research.
- All costs related to the research will be borne by the sponsor.
- My consent does not relieve the investigator and the sponsor of their responsibilities to me.

I freely and voluntarily agree to participate in the research proposed to me.

Attestation of oral consent of the patient

Prospective evaluation of the biomechanical properties of the thoracic aorta using magnetic resonance imaging and in-vitro elasticity tests in patients with ascending aortic aneurysms.

Study MECATHOR – 2018-A02010-55

(Done in 2 copies: one copy is given to the participant, the other is kept by the investigator)

I informed the patient of the objective and modalities of this research as well as the conditions of participation, his rights, expected benefits, constraints and foreseeable risks.

The patient read the information document that was explained to him and I gave him a copy.

The patient obtained the answers to the questions asked.

<table>
<tr><td colspan="2"><u>Oral consent :</u>

Patient surname and first name : ……………………………………………………….

Date of oral consent: ………/………/………</td></tr>
<tr><td>To be completed by the investigating physician

I, the undersigned, (surname-first name)……………………………………………………

declare that I have obtained the patient's oral consent to participate in this research.</td><td>Signature

Date ……/………/…….</td></tr>
</table>
