# Supplementary material for: Comparison of two techniques (in vivo and ex-vivo) for evaluating the elastic properties of the ascending aorta: Prospective cohort study
Source: PLoS One. 2021 Sep 13;16(9):e0256278. doi: 10.1371/journal.pone.0256278 (PMC8437267; doi:10.1371/journal.pone.0256278)
Supplement: S4 File — (PDF) [file pone.0256278.s004.pdf]

IM-2016-0369-002

Note d'information

Evaluation prospective des propriétés biomécaniques de l'aorte thoracique couplant imagerie par résonance magnétique et tests in-vitro d'élasticité chez des patients présentant un anévrisme de l'aorte ascendante

Etude MECATHOR

N° d'enregistrement : 2018-A02010-55

(Fait en 2 exemplaires : un exemplaire est remis au participant, l'autre est conservé par l'investigateur)

Promoteur : CHU Dijon Bourgogne – Délégation à la Recherche Clinique et à l'Innovation
1, Bd Jeanne d'Arc
BP 77908 - 21079 Dijon Cedex
Tél : 03 80 29 50 15 / Fax : 03 80 29 36 90

Madame, Mademoiselle, Monsieur,

Le Médecin Investigateur :

Titre, Nom, Prénom:
Nom du service:
Nom de l'établissement:
Adresse:
N° de téléphone:

et le CHU Dijon Bourgogne mènent actuellement une étude sur les anévrismes de l'aorte ascendante, coordonnée par le Dr. Marie-Catherine MORGANT du CHU Dijon Bourgogne.

Nous souhaitons vous solliciter pour participer à une **recherche interventionnelle à risques et contraintes minimes**, réalisée selon la loi n° 2012-300 du 5 mars 2012 relative à la politique de santé publique, modifiée par l'ordonnance n°2016-800 du 16 juin 2016 et ses décrets d'application, et définie au 2° de l'article L1121-1 du code de la Santé Publique.

Nous vous présentons ici les informations nécessaires pour comprendre l'intérêt et le déroulement de l'étude, les bénéfices attendus, les contraintes et les risques prévisibles.

Votre participation à cette recherche est entièrement volontaire et vous avez le droit de refuser d'y participer. Dans ce cas-là, vous continuerez à bénéficier de la meilleure prise en charge médicale possible, conformément aux connaissances actuelles.

Lisez attentivement cette notice, elle vous appartient. Vous pouvez la communiquer et en parler à votre médecin traitant ou à vos proches pour avis.
Posez toutes les questions qui vous sembleront utiles.
Après avoir obtenu les réponses satisfaisantes à vos questions et disposé d'un délai de réflexion adapté, vous pourrez alors décider si vous voulez participer à cette étude.

Pourquoi cette recherche ?

Un anévrisme de l'aorte thoracique est une dilatation localisée de la partie de l'aorte comprise entre sa naissance, au niveau cardiaque, et le diaphragme, c'est-à-dire le muscle horizontal qui sépare la cage thoracique de l'abdomen.
Bien que les mécanismes qui sous-tendent l'apparition d'un anévrisme soient assez bien connus, nous ne savons pas encore pourquoi certains anévrismes se rompent alors qu'ils ne sont encore que de petite taille (< 50 mm), alors que d'autres dépassent ce diamètre sans se rompre.
Le seul traitement curatif est chirurgical, avec remplacement de la portion dilatée par une prothèse. Actuellement, les recommandations chirurgicales sont basées sur le diamètre maximal de l'aorte ascendante (entre 45 et 55 mm selon l'existence d'éventuels facteurs de risques de rupture associés (maladie du tissu conjonctif, bicuspidie aortique, progression rapide de l'anévrisme)).

Quel est l'objectif de cette recherche ?

Pour mieux comprendre ces phénomènes, nous souhaitons étudier les propriétés élastiques de la paroi de l'aorte à partir des images de vos IRM cardiaques (traitement informatique des images). En parallèle, nous allons mesurer directement l'élasticité de l'aorte sur la pièce opératoire, c'est-à-dire sur la portion d'aorte malade que l'on va enlever et remplacer par une prothèse lors de la chirurgie (test ex-vivo).

→ *Déroulement de votre participation à cette étude :*

Consultation pré-opératoire ou arrivée dans le service de Chirurgie Cardiovasculaire et Thoracique pour une chirurgie de l'aorte ascendante

Information sur le principe et le déroulement de l'étude – Remise de la note d'information

↓ *Programmation de votre chirurgie*

Analyse des images d'IRM cardiaque pré-opératoire

Récupération des images de votre IRM cardiaque préopératoire et analyse d'images

↓

Chirurgie de l'aorte ascendante

Récupération de la pièce opératoire (paroi aorte anévrysmale) destinée au déchet
Réalisation des tests d'élasticité ex-vivo sur la pièce opératoire

↓

Fin de votre participation à l'étude

- Les IRM cardiaques sont des examens demandés en pratique courante avant et après une intervention sur l'aorte ascendante. Vous n'avez donc aucun examen d'imagerie à réaliser en plus.
- La paroi de l'aorte étudiée est une pièce opératoire c'est-à-dire qu'elle est normalement destinée au déchet une fois votre intervention terminée. En aucun cas, nous ne prendrons plus d'aorte que ce que votre pathologie impose. Les mesures d'élasticité seront réalisées sur 4 fragments prélevés sur cette pièce opératoire.
- Les analyses d'imagerie seront effectuées sur les examens d'IRM préopératoire que vous réaliserez dans le cadre de votre prise en charge normale.
- Les analyses d'élasticité sur la paroi de l'aorte seront réalisées exclusivement sur la pièce opératoire c'est-à-dire sur la partie de votre aorte destinée au déchet. Nous ne prélèverons pas plus de paroi que ce que votre pathologie nécessite.
- Dans le cadre de cette étude, seront recueillies des données épidémiologiques et médicales générales, des données d'imagerie IRM cardiaque préopératoire et postopératoire avec analyse spécifique d'élasticité et des données in-vitro de mesures d'élasticité de votre paroi aortique.

Quels sont les bénéfices et les risques possibles ?

Aucun bénéfice direct pour vous-même n'est attendu, mais il y a un bénéfice potentiel collectif à moyen terme selon les résultats. Si les mesures de compliance aortique par IRM s'avèrent corrélées aux mesures biomécaniques, cela permettrait une adaptation des indications opératoires en fonction des données imagerie spécifiques de la compliance de l'aorte propre à chaque patient. Cette étude ne présente par ailleurs pas de risque surajouté puisqu'il n'y a pas de geste supplémentaire par rapport à la pratique habituelle.

Quelles sont les modalités de prise en charge médicale ?

Cette recherche ne modifie à aucun moment les modalités de votre prise en charge et n'impose aucun suivi en fin de recherche ou en cas d'arrêt prématuré de la recherche, votre participation se terminant à la fin de votre premier suivi post-opératoire.

Le cadre réglementaire

Cette étude est réalisée selon la loi n° 2012-300 du 5 mars 2012, dite « Loi Jardé » relative à la politique de santé publique, modifiée par l'ordonnance n°2016-800 du 16 juin 2016 et ses décrets d'application, et définie au 2° de l'article L1121-1 du code de la Santé Publique.

Le promoteur a souscrit une assurance garantissant sa responsabilité civile pour couvrir les éventuelles conséquences préjudiciables de cette étude (compagnie SHAM, n° de contrat : 129.234, 18, rue Edouard Rochet, 69372 LYON Cedex 08).

Cette étude a reçu pour sa mise en œuvre un avis favorable du Comité de Protection des Personnes (CPP) Sud-Est IV, en date du 16/10/2018.

Protection de vos données

Cette étude sera réalisée conformément à la loi n°78-17 du 6 janvier 1978 relative à l'informatique, aux fichiers et aux libertés modifiée et au Règlement Général sur la Protection des Données personnelles (RGPD), adopté au niveau européen, et entré en application le 25 mai 2018.
Les données médicales et personnelles vous concernant ainsi que celles associées à vos prélèvements vont être traitées informatiquement afin d'établir les résultats de l'étude, conformément aux exceptions prévues à l'article 9 du RGPD permettant de traiter les données de santé.
Ce traitement se fera de manière **confidentielle** car vos données seront identifiées uniquement par vos initiales associées à un numéro de code. Elles seront transmises au Promoteur de la recherche et conservées pendant 15 ans.
Par ailleurs, en cas de retrait de consentement, vos données recueillies antérieurement seront utilisées et pourront être traitées dans les conditions prévues par la recherche.

Néanmoins, vous disposez de plusieurs droits que vous pouvez faire valoir par demande écrite auprès du Délégué à la Protection des Données – CHU Dijon Bourgogne – 1 Bd Jeanne d'Arc – 21079 DIJON Cedex : droit d'accès et de rectification de vos données, droit de limitation de leur traitement informatisé, droit d'opposition à leur transmission, droit à l'oubli (effacement de vos données), droit de réclamation auprès de la CNIL (Commission nationale de l'information et des libertés).

Vos droits quant à votre participation à cette étude :

- Vous avez la possibilité à tout moment de l'étude de contacter le médecin-investigateur pour toute demande d'information complémentaire sur l'étude, sur votre participation ou sur vos données personnelles liées à votre santé.

- Vous pourrez être informé(e), si vous le souhaitez, des résultats globaux de la recherche à la fin de l'étude auprès du médecin-investigateur.

- Les résultats de cette étude pourront faire l'objet de communications et/ou publications dans des revues scientifiques dans lesquelles, votre nom ne sera jamais utilisé.

Votre Participation est volontaire et libre

Votre éventuel refus de participer n'aura aucune conséquence sur le type et sur la qualité de votre prise en charge, ainsi que sur vos relations avec votre médecin. Si vous acceptez de participer, vous pourrez à tout moment quitter cette étude sans justification ni conséquence sur la qualité de votre prise en charge. Il vous suffit d'en informer votre médecin investigateur.

Nous vous remercions de votre coopération.
Si vous êtes d'accord pour participer à cette étude, nous vous demandons de bien vouloir donner votre accord oral.

Consentement Eclairé Exprès

Evaluation prospective des propriétés biomécaniques de l'aorte thoracique couplant imagerie par résonance magnétique et tests in-vitro d'élasticité chez des patients présentant un anévrisme de l'aorte ascendante

Etude MECATHOR

N° d'enregistrement : 2018-A02010-55

(Fait en 2 exemplaires : un exemplaire est remis au participant, l'autre est conservé par l'investigateur)

Le Médecin investigateur du service de chirurgie cardio-vasculaire et thoracique m'a proposé de participer à la recherche impliquant la personne humaine MECATHOR, dont le CHU DIJON BOURGOGNE est promoteur.

J'ai été informé(e) de l'objectif et des modalités de réalisation de cette recherche impliquant la personne humaine ainsi que de mes conditions de participation, de mes droits, des bénéfices attendus, des contraintes et des risques prévisibles, et **j'ai obtenu les réponses** aux questions que j'ai posées.

J'ai pris connaissance du document d'information qui m'a été expliqué et **j'en conserverai** un exemplaire.

Je déclare sur l'honneur être affilié(e) à un régime de sécurité sociale ou bénéficiaire d'un tel régime.

J'accepte pour des raisons liées à ma sécurité et pour le bon déroulement de la recherche impliquant la personne humaine :
- De répondre aux questions qui me seront posées à propos de mes antécédents médicaux et de suivre toutes les consignes et instructions qui me seront données par le médecin-investigateur ou son équipe, dont celles qui sont détaillées dans le document d'information.
- De contacter le médecin-investigateur ou son équipe dans les délais les plus brefs si je présente un événement anormal.

J'accepte également :
- Que l'ensemble de mon dossier médical soit consulté par les personnes habilitées dans le cadre de cette recherche.
- Le recueil des données médicales et personnelles décrites dans le document d'information ainsi que leur traitement informatique par le promoteur ou par des structures agissant pour son compte.

J'ai noté que :
- Les coordonnées du médecin-investigateur sont notées sur la note d'information qui m'a été remise.
- Ma participation à cette étude est volontaire et je peux à tout moment décider d'interrompre ma participation sans justification et sans que cela influence la qualité des soins que je recevrai. J'ai compris qu'en cas de retrait de consentement, mes données recueillies antérieurement pourront ne pas être effacées et pourront continuer à être traitées dans les conditions prévues par la recherche.
- L'intégralité des frais liés à la recherche seront pris en charge par le promoteur.
- Mon consentement ne décharge pas l'investigateur et le promoteur de leurs responsabilités à mon égard.

J'accepte librement et volontairement de participer à la recherche qui m'est proposée.

Attestation d'obtention du consentement oral du patient

Evaluation prospective des propriétés biomécaniques de l'aorte thoracique couplant imagerie par résonance magnétique et tests in-vitro d'élasticité chez des patients présentant un anévrisme de l'aorte ascendante

Etude MECATHOR

N° d'enregistrement : 2018-A02010-55

(Fait en 2 exemplaires : un exemplaire est remis au participant, l'autre est conservé par l'investigateur)

J'ai informé le patient de l'objectif et des modalités de réalisation de cette recherche ainsi que des conditions de participation, de ses droits, des bénéfices attendus, des contraintes et des risques prévisibles.

Le patient a pris connaissance du document d'information qui lui a été expliqué et **je lui en ai remis** un exemplaire.

Le patient a obtenu les réponses aux questions posées.

<table>
<tr><td colspan="2"><u>Consentement oral</u>

Nom-prénom du patient : ………………………………………………………..

Date de l'obtention du consentement oral : ………/………/………</td></tr>
<tr><td>A compléter par le médecin-investigateur

Je, soussigné(e), (nom-prénom) ………………………………………………………..

déclare avoir obtenu du patient le consentement oral à la participation à cette recherche</td><td>Signature

Le………/………/……….</td></tr>
</table>
